# Quasi-Dynamic Dissolution of Electrospun Polymeric Nanofibers Loaded with Piroxicam

**DOI:** 10.3390/pharmaceutics11100491

**Published:** 2019-09-24

**Authors:** Urve Paaver, Jyrki Heinämäki, Ivan Kassamakov, Tuomo Ylitalo, Edward Hæggström, Ivo Laidmäe, Karin Kogermann

**Affiliations:** 1 Institute of Pharmacy, Faculty of Medicine, University of Tartu, Nooruse 1, 50411 Tartu, Estonia; jyrki.heinamaki@ut.ee (J.H.); ivo.laidmae@ut.ee (I.L.); karin.kogermann@ut.ee (K.K.); 2 Electronics Laboratory, Department of Physics, P.O. Box 64 (Gustaf Hällströmin katu 2a), University of Helsinki, FI-00014 Helsinki, Finland; ivan.kassamakov@helsinki.fi (I.K.); tuomo.ylitalo@helsinki.fi (T.Y.); edward.haeggstrom@helsinki.fi (E.H.)

**Keywords:** wetting, in situ drug release, nanofibers, electrospinning, poorly water-soluble drug, piroxicam, hydroxypropyl methyl cellulose, polydextrose, scanning white light interferometry

## Abstract

We investigated and monitored in situ the wetting and dissolution properties of polymeric nanofibers and determined the solid-state of a drug during dissolution. Piroxicam (PRX) was used as a low-dose and poorly-soluble model drug, and hydroxypropyl methylcellulose (HPMC) and polydextrose (PD) were used as carrier polymers for electrospinning (ES). The initial-stage dissolution of the nanofibers was monitored in situ with three-dimensional white light microscopic interferometry (SWLI) and high-resolution optical microscopy. The physical solid-state characterization of nanofibers was performed with Raman spectroscopy, X-ray powder diffraction (XRPD), and scanning electron microscopy (SEM). We showed that PRX recrystallizes in a microcrystalline form immediately after wetting of nanofibers, which could lead to enhanced dissolution of drug. Initiation of crystal formation was detected by SWLI, indicating: (1) that PRX was partially released from the nanofibers, and (2) that the solid-state form of PRX changed from amorphous to crystalline. The amount, shape, and size of the PRX crystals depended on the carrier polymer used in the nanofibers and dissolution media (pH). In conclusion, the present nanofibers loaded with PRX exhibit a quasi-dynamic dissolution via recrystallization. SWLI enables a rapid, non-contacting, and non-destructive method for in situ monitoring the early-stage dissolution of nanofibers and regional mapping of crystalline changes (re-crystallization) during wetting. Such analysis is crucial because the wetting and dissolution of nanofibers can greatly influence the performance of nanofibrous drug delivery systems in pharmaceutical and biomedical applications.

## 1. Introduction

In recent years, advanced polymeric nanofibrous drug delivery systems (DDSs) have been of increasing use in pharmaceutical and biomedical applications. Such systems include oral dispersible thin films, ophthalmic preparations, multifunctional wound dressings, and implanted DDSs [1,2,3]. These hybrid polymer nanofibers hold great promise with respect to drug therapy and tissue engineering in both human and veterinary medicine. Nanofibers have a unique capability to modify material properties, which could have a paramount effect on drug delivery performance. They possess fiber structure dimensions at the nanoscale and a large outer surface-to-volume ratio, enabling multiple alternatives in drug delivery. The conversion of polymer solutions to nanofibers by solution electrospinning (ES) is affected by material, process, and ambient parameters [3,4,5]. The geometric and physicochemical properties of nanofibers depend, for example, on the nature of the solution, polymer type, polymer chain conformation, viscosity, elasticity, and electrical conductivity, as well as on solvent polarity and surface tension [4,5]. These structural fiber properties in turn can have a great impact on the dissolution and drug release of fibers [6,7,8,9,10,11]. To date, however, our understanding on the dissolution (drug release) behavior of polymeric nanofibers and related physicochemical mechanisms is still limited. This is partially due to the fact that the nanofibers are challenging samples for analysis due to their nanoscale size and brittleness.

In the state-of-the art literature there are many examples of how the geometric nanofiber properties (size, size distribution, surface morphology, orientation) and porosity can greatly affect the dissolution (drug release) and therapeutic efficiency of nanofibrous DDSs [6,12,13,14]. With poorly water-soluble drugs, the limitations in drug release of the nanofibrous DDSs can delay the onset of drug action, cause poor oral bioavailability, and ultimately decrease therapeutic effect. The geometric nanofiber properties can be governed by the adjustment and control of the critical material, process, and environmental parameters in an ES process [5,15,16]. Lately, increasing interest has been focused on developing ES processes which could produce drug-loaded nanofibers of a specific quality and with reproducible drug release. Due to the nanoscale size, controlling the quality and performance of such nanofibers is still challenging. Consequently, the mechanisms of drug release associated with such novel polymeric nanofibrous DDSs remain poorly understood.

Scanning white light microscopic interferometry (SWLI) is a high-depth resolution imaging technique suitable for determining the geometric properties (i.e., fiber shape, diameter, and length), porosity, and surface topography of nanofibrous DDSs [17]. Three-dimensional (3D) SWLI permits rapid, non-contacting and non-destructive imaging of nanofibrous samples, since it requires neither sample preparation nor modification. Recently, we applied SWLI and high-resolution scanning electron microscopy (SEM) equipped with a customized measurement program for 3D surface topography analysis of non-woven nanofibrous mats [17]. In pharmaceutical research, the use of SWLI for investigating and non-contact imaging of the manufacturing processes and final products is still limited. Sandler et al. and Genina et al. used SWLI to image 3D printed multi-layered DDSs [18,19]. Hanhijärvi et al. applied SWLI to investigate the film surface properties of hydroxypropyl methylcellulose (HPMC)-coated tablets [20], while O’Bryan et al. used it to image 3D printed microgels intended for bioprinting applications [21]. Recently, Wickström et al. applied SWLI to characterize the surface texture and surface roughness of antibiotic-loaded fiber-reinforced composite implants [22]. 

The aim of the present study was to provide insight into the wetting and early-stage dissolution of drug loaded polymeric nanofibers. To gain an understanding of such phenomena, we monitored in situ the wetting and drug release of fibers loaded with a poorly water-soluble drug (piroxicam, PRX), and determined the solid-state of the drug during dissolution. Two hydrophilic polymers were investigated in nanofibers: hydroxypropyl methylcellulose (HPMC) intended for the sustained drug release and polydextrose (PD) intended for the immediate drug release of a poorly water-soluble drug [23,24]. We combined SWLI and high-resolution optical microscopy to image PRX-loaded nanofibers in contact with an aqueous medium, and the physical appearance and solid-state changes of nanofibers were verified with scanning electron microscopy (SEM), Raman spectroscopy, and X-ray diffraction (XRD), respectively. To our knowledge, this is the first time when such imaging and solid-state analyses are applied in a complimentary fashion to investigate the dissolution of polymeric nanofibers. 

## 2. Materials and Methods 

### 2.1. Materials

**Figure 1:**
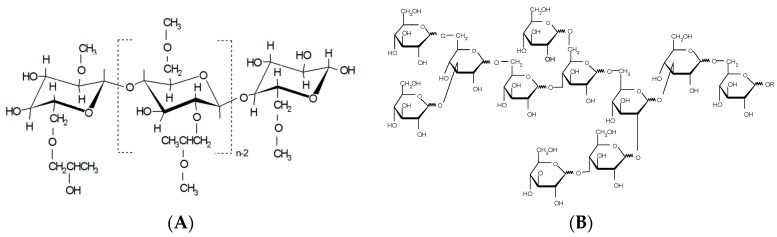
Structural formula of (**A**) hydroxypropyl methylcellulose (HPMC) and (**B**) polydextrose (PD).

Commercially available cellulose ether, hydroxypropyl methylcellulose, HPMC (Methocel K100M premium CR, Colorcon Ltd., Dartford, Kent, UK), and polydextrose, PD (STA-LITE L90, 70% aqueous solution of PD, Tate & Lyle Netherlands B.V., Koog Aan De Zaan, KA, Netherlands) (Figure 1) were used as carrier polymers for the ES of nanofibers. The organic solvents were 1,1,1,3,3,3-hexa-fluoro-2-propanol (HFIP) (≥99.0%) (Sigma-Aldrich C.C., St. Louis, MO, USA) and methanol (Lach-Ner, s.r.o., Neratovice, Czech Republic). Anhydrous piroxicam, (PRX, pure form I, PRXAH I, Letco Medical, Inc., N Livonia, MI, USA) was selected as a model drug for a low-dose poorly water-soluble active substance in nanofibers. In the wetting and dissolution experiments, purified water, hydrochloric acid buffer solution, USP 28 (pH 1.2), and phosphate buffer solution (pH 7.2) were used as dissolution media. The materials for the preparation of buffer solutions were of analytical grade and purchased from Lach-Ner, s.r.o., Neratovice, Czech Republic.

### 2.2. Preparation of Fiber Mats 

The carrier polymer (HPMC) and model drug (PRX) were first manually dry mixed with a mortar and pestle at three different ratios (1:1, 1:2, and 1:4). Next, the pre-mixtures were dissolved in organic solvent to obtain the 0.8% (*w*/*V*) solution of HPMC in HFIP as described in our previous study [16]. With PD, the aqueous solution of PD (70%) alone or the mixture of the aqueous solution of PD (70%) and methanol (5:1) were used for ES. The amount of PRX in the 70% aqueous solution of PD (0.75 mL) used for ES was 20 mg, and 0.75 mL contained 530 mg PD. The drug-loaded nanofibers were fabricated with an in-house ES set-up equipped with a syringe system, automatic syringe pump, spinneret, high-voltage power supply, and a grounded collector plate. The high-voltage power supply system was a Gamma (Gamma High Voltage Research, Model ES3OP-10W/DAM, Ormond Beach, FL, USA). For HPMC solutions, the ES distance and voltage between the spinneret and the collector plate were 8.0 cm and 7 kV, respectively. The pump rate of the automatic syringe pump (KdScientific, Model No: KDS-250-CE, Geneq Inc, Montreal, Quebec, Canada) was 1.0 mL/h. For PD solutions, the ES distance, voltage, and pump rate were 17 cm, 14 kV, and 2.0 mL/h, respectively. The prepared fibers were stored in desiccators (above silica gel, with relative humidity (RH) of 0%) at ambient room temperature (21 ± 2 °C) and in a refrigerator (6 ± 2 °C/RH 0%) prior to use. 

### 2.3. Physical Appearance and Solid-State Characterization 

The size and surface morphology of fibers were investigated with scanning electron microscopy, SEM (Helios NanoLab 600, FEI Company, Hillsboro, OR, USA). The samples were coated with a 3-nm gold layer in an argon atmosphere using a magnetron sputter prior to SEM imaging. Solid-state characterization of the starting materials and the electrospun fibers were carried out with X-ray diffraction, XRD (D8 Advance, Bruker AXS GmbH, Karlsruhe, Germany), and Raman spectroscopy (B&W Tek Inc., Newark, DE, USA). In XRD, Cu Kα1 radiation (λ= 1.5418 Å, 40 kV, and 40 mA) was used, and data were collected in the range of 5° and 35° 2*θ* with a step size of 0.2° 2*θ*. The brittleness of the fibrous mats was detected by visual inspection and manual palpation with tweezers.

### 2.4. In Situ Wetting and Dissolution Tests of Fibers 

For in-situ wetting tests, we applied the fiber mats as such and the tablets compressed from these fibers. The initial-stage dissolution of the fiber mats were monitored by a high-resolution optical microscopy (Microscope Leica DMLB equipped with the Leica Germany 5.0× and L50×/0.50 objectives and Canon Power Shot 550 digital camera). A standardized drop (50 μL) of aqueous dissolution media was gently placed in contact with a fiber mat at room temperature (21 ± 2 °C), and the dissolution was monitored by taking micrographs at regular intervals. 

Pieces of a fiber mat were manually compressed into small flat-faced tablets (discs) with a miniaturized press (Specac Ltd., Orpington BR5 3FQ, UK) intended for preparing sample discs for infrared (IR) spectroscopy analysis. The compression pressure was 196 MPa (2t/cm^2^) and the compression time was 5 s. The tablets (discs) were 12.7 mm in diameter and 0.8–1.0 mm in thickness. For in situ initial-stage dissolution experiments, one micro-pipette drop of purified water (2 µL) was gently placed onto the surface of fibrous tablets at room temperature (21 ± 2 °C). The contact point of the drop onto the tablet surface was kept as constant as possible.

**Figure 2:**
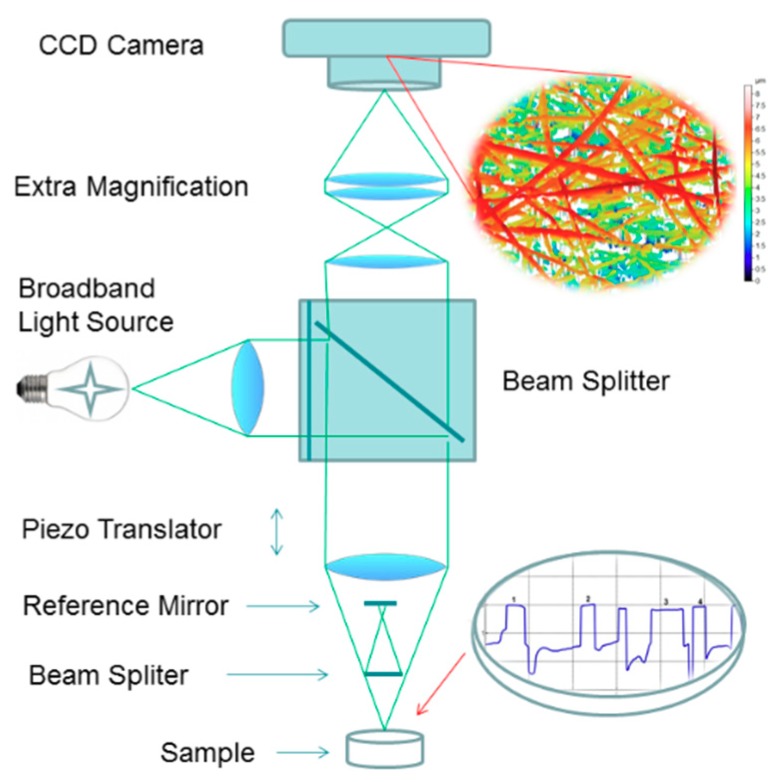
Schematic diagram of scanning white light interferometer (SWLI) and the experimental setup for the in situ wetting and early-stage dissolution of nanofibers.

The wetting and early-stage dissolution of fibrous tablets were monitored in situ with an in-house 3D scanning white light microscopic interferometry (SWLI). In SWLI, a light beam passes through an interferometric objective (Nikon, Michelson type, magnification 120×) containing a beam splitter that reflects half the incident beam onto a reference surface and passes the other half onto the test surface (Figure 2). Light reflected from the test and reference surfaces recombines and interferes to form an interferogram. The working principle and structure of SWLI is described elsewhere. For details see Kassamakov et al. [25,26], Hanhijärvi et al. [20], and Paaver et al. [17]. In the present study, we obtained three-dimensional (3D) images featuring 29 nm × 29 nm active pixel size from a 55 × 40 μm^2^ area. The time period of 10 min prior to a subsequent drop was required for the complete SWLI analysis of the tablets. Purified water (pH 5.6) and the USP 28 buffer solutions (hydrochloric acid buffer pH 1.2 and phosphate buffer solution pH 7.2) were used as dissolution media. The total number of optical microscopy and SWLI images taken on the course of the in situ dissolution experiments was over 500 (including short video-clips). The number of samples was 3–6 in each analysis.

A high-resolution scanning electron microscope (SEM) (Helios NanoLab 600, FEI Company, Hillsboro, OR, USA) was used as a reference method to the SWLI. A software-based measurement function xT Microscope Control (FEI) was used to measure the horizontal dimensions of the nanofibers. Samples were mounted on aluminum stubs with silver paint and were magnetron-sputter coated with a 3-nm gold layer in an argon atmosphere prior to microscopy. ImageJ (vers. 1.50i) was used for fiber size distribution analysis.

### 2.5. Dissolution Test

The in vitro dissolution tests of the electrospun nanofiber mats and the pure PRX powder samples were performed using an USP dissolution apparatus I (basket method) in a semi-automated dissolution system (Termostat-Sotax AT7, Sotax AG, Aesch, Switzerland). The concentration of PRX in the dissolution medium was measured at 354 nm by using a UV-VIS spectrophotometer (Ultrospec III, Biochrom Ltd, Cambourne, Cambridge, UK). The dissolution medium was 900 mL of purified water (pH 5.6) and buffer solution (pH 1.2 or pH 7.2) maintained at 37 ± 0.5 °C as described in the USP 28. The basket rotation speed was 50 rpm. The amount of PRX in the powder and nanofiber samples used in the dissolution test was 20 mg. Six parallel tests were performed for each sample.

## 3. Results and Discussion

### 3.1. Physical Appearance of Nanofibers

**Figure 3:**
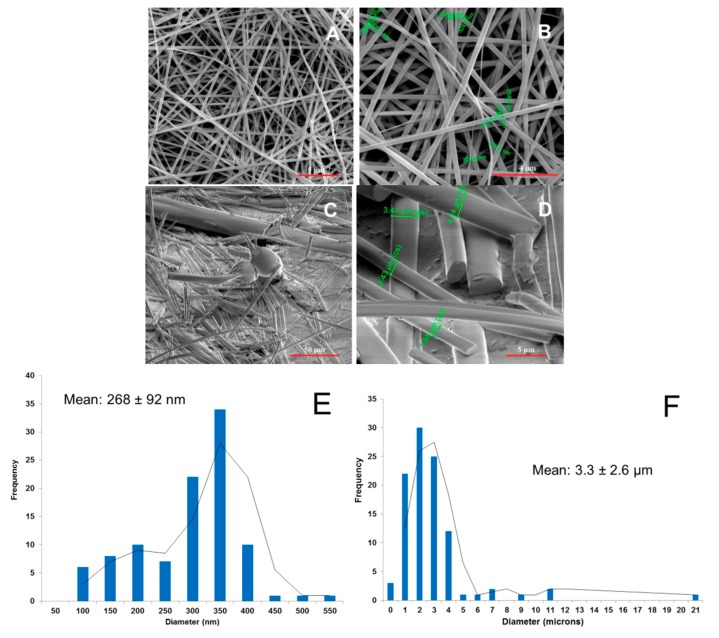
SEM images of piroxicam (PRX)-loaded hydroxypropyl methylcellulose (HPMC) (**A,B**) nanofibers and polydextrose (PD) (**C,D**) fibers. Magnification of SEM: 5000× (**A**), 10000× (**B**), 600× (**C**), and 5000× (**D**). (**E**) Fiber size distribution of the PRX-loaded HPMC nanofibers; (**F**) Fiber size distribution of the PRX-loaded PD fibers.

The ES of PRX-loaded fibers with both HPMC and PD polymers resulted in yellowish and white thin fiber mats with a porous internal structure, respectively (Figure 3). HPMC generated fibrous mats with a nanoscale fiber diameter ranging from 100 nm to 400 nm and uniform fiber thickness (Figure 3A,B,E). With PD, we found that due to the hygroscopic nature of PD, the ES of PRX-loaded fibers was possible only in precisely controlled and optimized temperature and humidity conditions (below 30 °C and 40% RH). The PD fiber mat consisted of individual short fibers reaching micron size and a large spread in diameters (Figure 3C,D,F). The diameter of these fibers ranged from 400 nm to 5 µm, and the corresponding fiber mats were brittle (based on palpation with tweezers). A number of spherical beads were observed (SEM) in the electrospun PD fiber mats (Figure 3C). This was due to the high surface tension and low viscosity of the solution. The fiber mats prepared from the mixtures of an aqueous PD solution and methanol, were smaller in diameter, more uniform in radial size, and less brittle (based on visual inspection and palpation with tweezers). The thickness of an entire PD fiber mat ranged from few microns to several tens microns (SWLI).

### 3.2. Physical Solid-State Properties of Nanofibers

**Figure 4:**
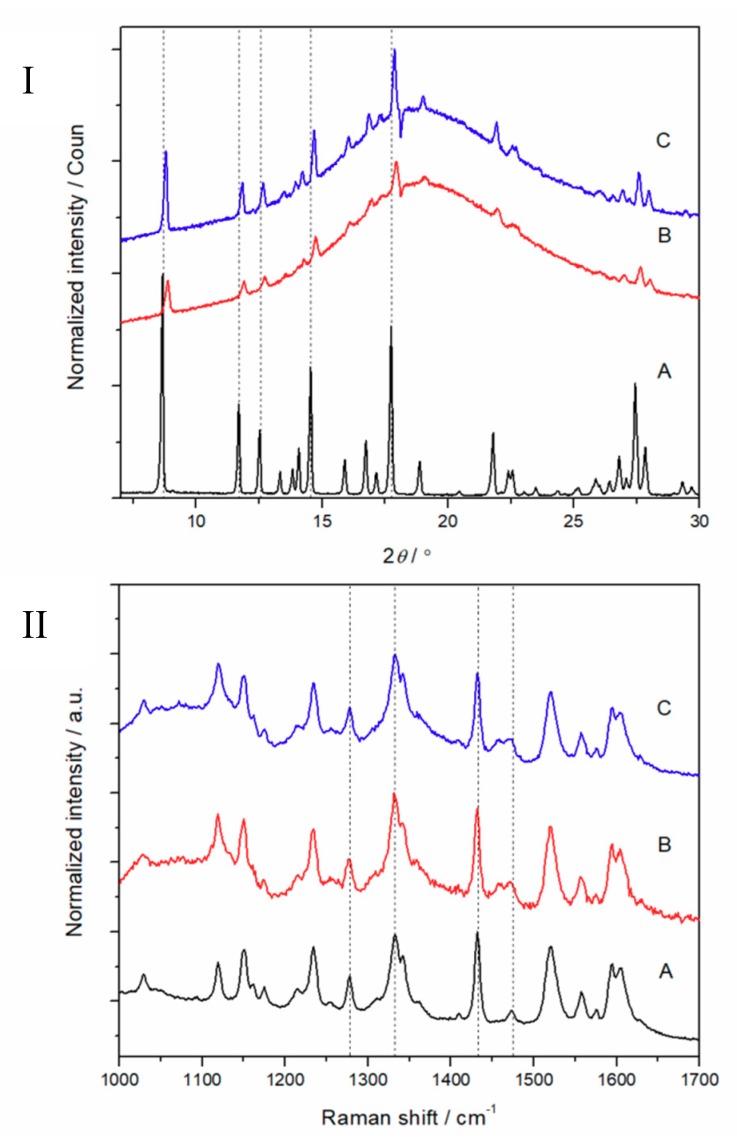
XRD patterns (**I**) and Raman spectra (**II**) of piroxicam (PRX)-loaded polydextrose (PD) fibers. Key: (**A**) PRX anhydrate form I (PRXAH I); (**B**) Fibers immediately after preparation; (**C**) Fibers stored for 30 days at 0% room humidity (RH) and low temperature (6 °C).

Recently, we reported that the electrospun HPMC nanofibers loaded with PRX are amorphous, and they remain as an amorphous form during a short storage period [16]. In the present study, we found that the ES of PD fibers loaded with PRX did not generate the corresponding amorphous structure, or the produced PRX amorphous structure was very unstable and immediately recrystallized as also discussed previously [27]. The solid-state properties of the electrospun PD fibers loaded with PRX were investigated immediately after the preparation and after a 1-month short-term storage at 0% RH and low temperature (6 °C). As seen in Figure 4 (I), the XRD patterns of electrospun fiber mats showed an amorphous halo together with the characteristic crystalline reflections of PRX anhydrate form I (PRXAH I) immediately after preparation. The XRD and Raman spectroscopy results (Figure 4 (II)) confirmed that PRX (PRXAH I) had lost some of its crystallinity during ES, but did not stay amorphous. No other crystalline reflections (XRD) or spectral features (Raman) characteristic to the known anhydrous PRX solid-state forms were observed in XRD diffractograms or Raman spectra.

### 3.3. In Situ Wetting and Initial Dissolution of Fibers 

**Figure 5:**
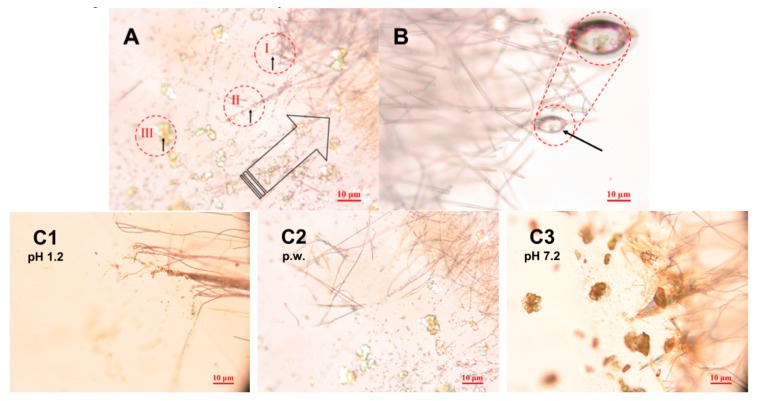
(**A**) Three phases identified in the release and crystallization of piroxicam (PRX) from hydroxylpropyl methylcellulose (HPMC) nanofibers. High-resolution light microscopy image (magnification 50×) validates the results obtained with SWLI (shown in Figure 6): **I**. PRX embedded in the nanofibers. **II**. Crystallization inside or on the nanofibers. **III**. Recrystallization of PRX. The large arrow indicates the propagation direction of the water front. Magnification 50×. (**B**) Representative micrograph on the initial dissolution of PRX–loaded polydextrose (PD) fibers. Visible crystals of PRX evident (highlighted with a black arrow) inside a red circle. Magnification 50×. (**C**) The PRX crystals visible at 30 s after wetting of nanofibers (HPMC:PRX 1:4) by placing a small drop of different dissolution media onto the surface of the fibrous sample: (**C1**) hydrochloric acid buffer solution pH 1.2; (**C2**) purified water, p.w. (pH 5.6); (**C3**) phosphate buffer solution pH 7.2 (22 ± 2 °C). Magnification 50×.

**Figure 6:**
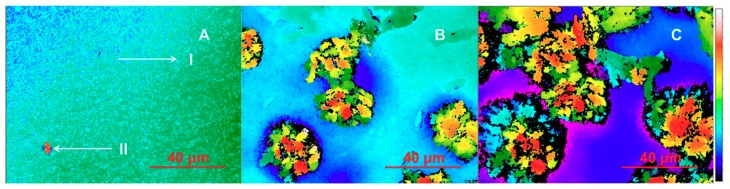
Time-lapse of wetting induced piroxicam (PRX) crystallization on the fixed surface of the tablet consisting of PRX-loaded hydroxypropyl methylcellulose (HPMC) nanofibers. The PRX release is visualized by scanning white light interferometry (SWLI). Key: (**A-I**) Initial state of the tablet; (**A-II**) After wetting with 2 µL of water and evaporating for 1 min; (**B**) After wetting a second time with 2 µL of water and evaporating for 10 min; (**C**) After wetting a third time with 2 µL water and evaporating for 10 min. The scale for height differences is changed according to changes in surface altitude (A—0 to 8 μm; C—0 to 90 μm). The diameter and thickness of tablet (disc) are 12.7 mm and 0.8–1.0 mm, respectively. Magnification 30×.

Figure 5 and Figure 6 show the high-resolution optical microscopy and SWLI results on the initial stages of the release and dissolution of PRX from the HPMC and PD fiber mats. Since PRX was completely dissolved in the HPMC solution, it is evident that the drug is homogeneously dispersed within nanofibers after ES, and hence the release would be uniform from fiber mats. Figure 5A shows a time-lapse-like snapshot from a high-resolution optical microscope of what happens to the PRX-loaded HPMC nanofiber mats during wetting and initial-stage dissolution. As shown in Figure 5A, the polymeric HPMC-PRX nanofibers dissolved within 1–5 s in the aqueous dissolution media used. According to the literature, PRX exists in an amorphous form in electrospun HPMC nanofibers [16,17,28,29], and the amorphous state of PRX hastened the onset of wetting and premature drug release. In our study, in situ wetting and dissolution monitoring indicated that PRX recrystallizes in a microcrystalline form immediately after wetting and release from the HPMC nanofibers. The PRX microcrystals ranging from 0.2 μm to 12 μm in size were formed after the addition of a standardized drop (50 μL) of purified water, hydrochloric acid (pH 1.2) or phosphate buffer (pH 7.2) solutions onto the HPMC nanofiber mats (Figure 5A,C). The recrystallization of PRX was associated with the formation of larger clusters of microcrystals. 

**Figure 7:**
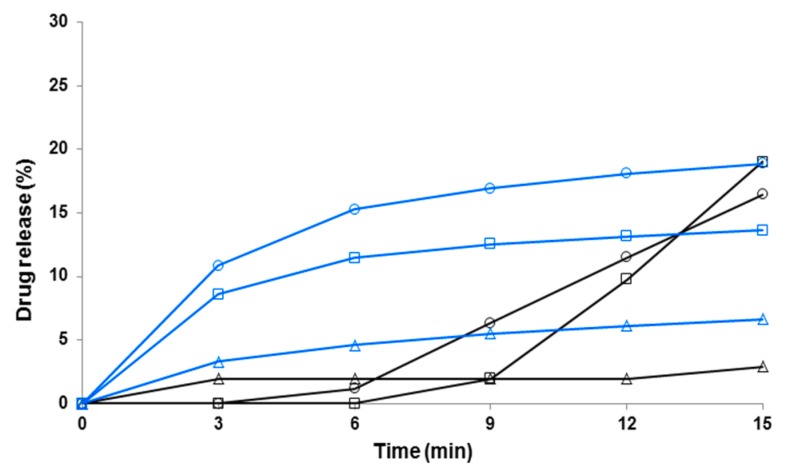
Early-phase dissolution of piroxicam (PRX)-loaded hydroxypropyl methylcellulose (HPMC) nanofibers freely set into the dissolution baskets (*n* = 6). The ratio of a carrier polymer (HPMC) and PRX is 1:1 in the nanofibers. Key: black curve—PRX anhydrate form I (PRXAH I) in a powder form; blue curve—HPMC:PRX nanofibers 1:1; Δ—purified water; ○—pH 7.2; □—pH 1.2. Standard deviation (SD) in each time point is less than 0.5%.

**Figure 8:**
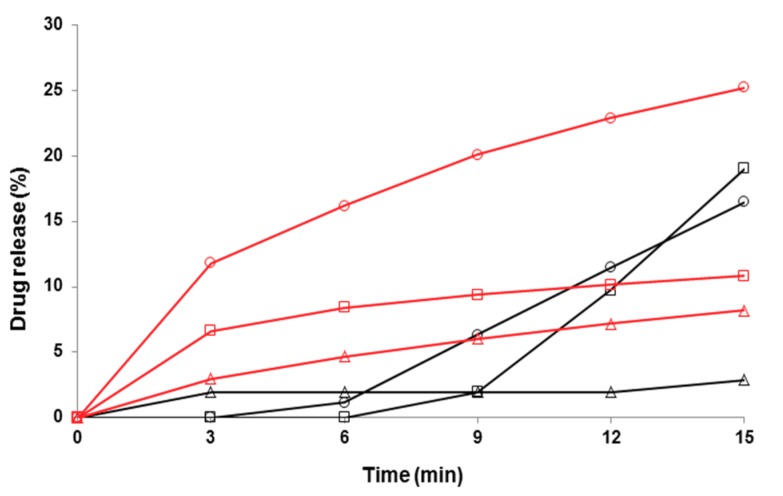
Early-phase dissolution of piroxicam (PRX)-loaded hydroxypropyl methylcellulose (HPMC) nanofibers freely set into the dissolution baskets (*n* = 6). The ratio of a carrier polymer (HPMC) and PRX is 1:4 in the nanofibers. Key: black curve—PRX anhydrate form I (PRXAH I) in a powder form; red curve—HPMC: PRX nanofibers 1:4; Δ—purified water; ○—pH 7.2; □—pH 1.2. Standard deviation (SD) in each time point is less than 0.5%.

**Figure 9:**
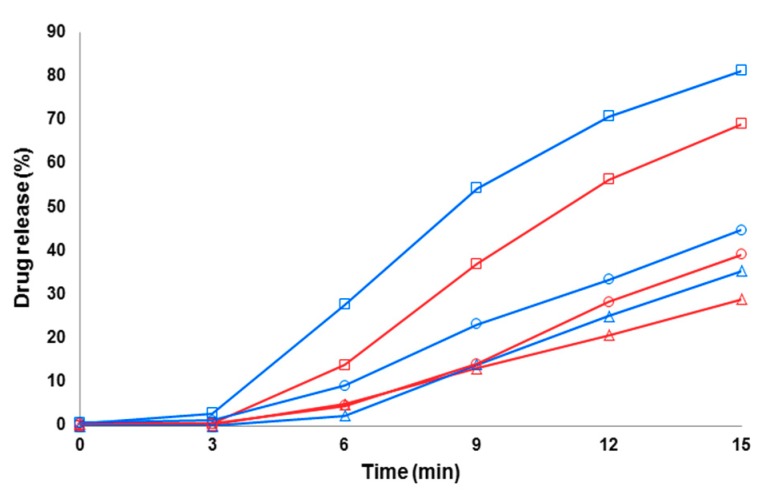
Early-phase dissolution of piroxicam (PRX)-loaded polydextrose (PD) fibers freely set into the dissolution baskets (*n* = 6). Key: red curve—the fibers containing PRX anhydrate form I (PRX AH I) and PD (electrospun from water solution); blue curve—the fibers containing PRXAH I and PD (electrospun from a water-in-methanol 5:1 solution); Δ—purified water; ○—pH 7.2; □—pH 1.2. Standard deviation (SD) in each time point is less than 1.4%.

The overall initial-stage dissolution behavior of freely water-soluble PD fibers loaded with PRX was similar, but faster than that observed with the corresponding HPMC nanofibers. As seen in Figure 5B, individual PRX crystals entrapped inside the PD fibers, and the release and dissolution of PRX from the PD fibers progressed via crystal formation. The rapid dissolution of a carrier polymer (PD) fostered the dissolution of PRX. The number and size of the PRX crystals differed from those observed with HPMC nanofibers. With PD, the number of crystals detected was smaller and the size of the crystals slightly larger (ranging from 0.3 μm to 15 μm) than with the HPMC nanofibers. The PRX crystals were located (clustered) inside the PD fibers (Figure 5B), which evidently could advance the dissolution of PRX since PD is a freely water-soluble carrier material. Consequently, this is an interesting approach to use freely water-soluble carrier polymer in electrospun fibers for the improvement of drug dissolution rate. The dissolution behavior of both types of drug-loaded fibers was verified with an established in vitro dissolution test (USP), and the results are shown in the next section (Figure 7, Figure 8 and Figure 9). 

Figure 5C illustrates the status of the HPMC:PRX (1:4) nanofibers at 30 s after wetting the surface of a thin nanofibers sample layer by a small drop (2 µL) of different dissolution media (hydrochloric acid buffer solution at pH 1.2, purified water, or phosphate buffer solution at pH 7.2). The number, shape, and size of the PRX crystals depended on the pH of the dissolution media used. With all dissolution media, the dissolution of nanofibers occurred via immediate crystal formation of PRX (i.e., recrystallization to microcrystals). Rapid changes in the solubility of PRX were also observed with a high-resolution optical microscope. The largest PRX microcrystals and clusters (ranging from few micrometers up to 10 µm) were present with phosphate buffer solution at pH 7.2 (Figure 5C3) and the smallest (0.2–1 µm in size) with hydrochloric acid buffer solution at pH 1.2 (Figure 5C1). It is evident that the recrystallization of PRX associated with the dissolution of HPMC nanofibers is dependent on both the pH and viscosity of dissolution media (in the microenvironment of dissolving nanofibers). Based on our findings, the lower pH of the dissolution media (pH 1.2) induces PRX to recrystallize into small 0.2–1 µm microcrystals in the vicinity of dissolving HPMC nanofibers. In addition, the subsequent formation of microcrystalline clusters occurred slower than that observed with the corresponding nanofibers in the higher pH of dissolution media (pH 7.2). The release of drug from nanofiber mat is shown to be also dependent on the mat thickness [30]. In the present study, the thickness of the HPMC fiber mat was in the range of 2–3 µm (measured by SEM and SWLI).

The SWLI images on the surface of the tablets compressed from the drug-loaded HPMC nanofibers are presented in Figure 6. Figure 6A shows a SWLI image of a tablet surface, where individual fibers are visible: A(I) initial state of tablet (magnification 30×) and A(II) after wetting with 2 µL of water and evaporating for 1 min. In the initial stage, the tablet surface is flat and there is no crystal formation visible. This indicates that PRX is located in nanofibers and most likely is still amorphous. Point A(II) is the first wetting point after adding 2 µL of water and evaporating for 1 min. Figure 6B illustrates the surface of the same tablet after wetting with another 2 µL of water and evaporating for 10 min. Initiation of crystal formation is visible indicating that PRX is partially released from the nanofibers (as seen in Figure 6B). This indicates also that the solid-state form of PRX has changed from amorphous to crystalline form. In contact with water, PRX anhydrate form I transfers to PRX monohydrate before dissolving [27]. It is obvious that PRX monohydrate crystals are visible in SWLI images (Figure 6B,C). Figure 6C shows that the surface of the same tablet after the identical wetting procedure applied to the same spot for a third time. The release of PRX and crystallization occur rapidly, and most likely the crystals of PRX monohydrate are instantly formed in the vicinity of nanofibers. This in turn leads to the retarded quasi-dynamic dissolution of PRX. The present finding is in good agreement with our previous study describing the dissolution behavior of PRX anhydrate and monohydrate forms [31].

In summary, the three phases of the initial-stage dissolution of PRX-loaded HPMC nanofibers were observed with the present compressed tablets and with the corresponding free nanofiber mats studied by SWLI and high-resolution optical microscopy, respectively. These phases are as follows (as shown in Figure 5 and Figure 6): (A) PRX is embedded in the nanofibers (in an amorphous state confirmed by our earlier study [16]), and all nanofibers are intact prior to the water front moving into them; (B) Crystallization of PRX starts inside the nanofibers; (C) Recrystallization of PRX is finalized after drug release from nanofibers.

### 3.4. Dissolution of Fibers 

Figure 7, Figure 8 and Figure 9 show the early-phase dissolution curves for the PRX anhydrate form I (PRXAH I) in a powder form, the PRX-loaded HPMC nanofibers, and PRX-loaded PD fibers. PRXAH I in a powder form exhibited clearly a longer lag-time in the early-phase dissolution than that observed with the drug-loaded HPMC or PD fiber mats. The early-phase dissolution of PRX anhydrous form I powder was also dependent on the dissolution media used (the amount of PRX dissolved in purified water within the first 15 min was only less than 3%). The present results are also supported by the results on the dissolution behavior of PRX obtained in our previous study [31]. 

As shown in Figure 7 and Figure 8, the HPMC nanofibers accelerated the immediate dissolution of PRX compared to that obtained with pure PRX anhydrous form I in a powder form. With the drug-loaded HPMC nanofibers, the early-phase dissolution of PRX was fast within the first 3 min followed by a sustained-release phase (Figure 7 and Figure 8). This accelerated dissolution is obviously due to an immediate release of amorphous PRX from the surface of nanofibers and formation of PRX microcrystals in the vicinity of nanofibers (as shown in Figure 5A). The initial drug release from the HPMC nanofibers depended on the dissolution medium, and this finding was in line with the optical microscopy images on the dissolution of HPMC nanofiber mats shown in Figure 5C. The subsequent sustained dissolution phase of PRX observed with the HPMC nanofibers (Figure 7 and Figure 8) is most likely partially due to the gel formation of HPMC as the nanofibers are dissolved, thus forming a hydrophilic gel barrier for the release of drug.

With the drug-loaded PD fibers, the initial-stage dissolution of PRX within the first 3 min was somewhat slower than that obtained with the PRX-loaded HPMC nanofibers (Figure 9). This is obviously due to the crystalline state PRX in the PD fibers, thus resulting in a short delay in the onset of dissolution. In spite of a 3-min lag-time, over 70% of PRX was dissolved within 15 min in acidic dissolution medium (pH 1.2) when formulated as PD fibers. The use of methanol as a co-solvent for ES slightly improved the dissolution of PRX from the PD fibers compared to the corresponding fibers electrospun with pure aqueous solution of PD (i.e., without any organic solvent component) (Figure 9). 

## 4. Conclusions

A solution electrospinning (ES) can be used to prepare composite hydroxypropyl methyl cellulose (HPMC) and polydextrose (PD) nanofibers loaded with piroxicam (PRX). The wetting and early-phase dissolution of PRX from these fiber mats are accelerated compared to PRX anhydrate form I in a powder form. With PRX-loaded HPMC nanofiber mats, the dissolution of drug progressed via immediate wetting of the polymeric matrix followed by a lucid recrystallization and dissolution of the drug. A poorly water-soluble PRX recrystallizes in a microcrystalline form after wetting of nanofiber mats, and the further dissolution of PRX is dependent on the pH of the dissolution media. The accelerated dissolution of PRX from freely water-soluble PD nanofibers occurs also via immediate microcrystals formation after wetting. We propose that the present electrospun nanofibers mats loaded with PRX exhibit a quasi-dynamic dissolution behavior. SWLI provides rapid non-contacting and non-destructive in situ monitoring of early-stage dissolution of nanofibers and regional mapping of crystalline changes (re-crystallization) during wetting.
